# Identification of origin and runoff of karst groundwater in the glacial lake area of the Jinsha River fault zone, China

**DOI:** 10.1038/s41598-022-18960-9

**Published:** 2022-08-29

**Authors:** Jianfei Ma, Xiangquan Li, Chunchao Zhang, Changchang Fu, Zhenxing Wang, Zhanxue Bai

**Affiliations:** 1 Institute of Hydrogeology and Environmental Geology, CAGS, Shijiazhuang, 050061 Hebei China; 2 Key Laboratory of Groundwater Sciences and Engineering, Ministry of Natural Resources, Shijiazhuang, 050061 Hebei China

**Keywords:** Environmental sciences, Hydrology

## Abstract

Karst groundwater plays important roles as a water supply and in sustaining the biodiversity and ecosystems of the eastern Qinghai–Xizang Plateau. Owing to the stratigraphic structure, high tectonic activity, and changeable climate of the region, the recharge source, runoff path, and dynamic characteristics of karst groundwater are highly complex, which poses challenges with regard to the protection of water resources and ecology. This study identified the origin and flow processes of karst groundwater in the glacial lake area of the Jinsha River fault zone using satellite remote sensing, hydrochemical and isotope analyses, and flow measurements. Results showed that active faults control the distribution of glacial lakes and the recharge, runoff, and discharge of karst groundwater. Glacial lake water is an important source of karst groundwater in the Jinsha River fault zone area. Specifically, glacial lake water continuously recharges the karst system via faults, fractures, and karst conduits, thereby maintaining the relative stability of karst spring flows. Through hierarchical cluster analysis, two main runoff conduits of karst water were distinguished: one along the Dingqu Fault and the other along the Eastern Zhairulong Fault, which together account for 59% of the total regional karst groundwater flow. The elevation difference between the recharge and discharge areas of the main karst springs is > 1000 m. Groundwater runoff is fast and residence time in the aquifer is short. The dissolution of calcite and dolomite mainly occurs during transit through the groundwater system, and cation exchange is weak. Therefore, the regional karst springs are predominantly HCO_3_−Ca·Mg type. To protect regional karst water resources and ecology, the monitoring and protection of glacial lakes should be strengthened.

## Introduction

The hydrological systems operating in karst catchments are highly complex, and karst aquifers show strong spatial heterogeneity^1–5^. The role and impact of specific hydrological processes under different hydrological conditions are often difficult to quantify, and the high heterogeneity of karst aquifers also poses a challenge with regard to related research^6^. Owing to the dense development of geological structures and transformation attributable to glaciation, research on the karst hydrological system in the Jinsha River fault zone on the eastern Qinghai–Xizang Plateau is difficult^7,8^. Because karst groundwater is important as a resource not only for human society but also for supporting the fragile ecological environment in this region, it is important to understand the karst groundwater circulation in the glacial lake area of the Jinsha River fault zone.

The karst hydrological system in the Jinsha River fault zone has a number of distinctive characteristics. (1) A complex geological structure. The Jinsha River fault zone is located at the junction between the Indian Plate and the Eurasian Plate. It is the region with the highest rates of geological activity and the most complex geological structures in the world^8–10^. Active geological structures, dense active faults with complex lithological combinations and spatial distributions exacerbate the complexity of the spatial structure of soluble rock strata in this area. (2) Multiple ranges of elevation of developed karst conduits. The initial lifting of the Qinghai–Xizang Plateau began in the Eocene, following which it rose rapidly during the late Pliocene to the Pleistocene^11^. A short hiatus during the overall lifting process allowed karst conduits to become well developed at multiple elevation ranges, some of which are connected by active faults^12^, which further aggravates the complexity of the karst hydrological system. (3) Diverse supply sources. Glacial lakes are distributed at higher elevations^13^. On the one hand, studies have shown that glacial lakes distributed at high elevation could be groundwater recharge sources^4,14–17^; on the other hand, there has been no systematic study on the distribution and genesis of glacial lakes near the Jinsha River fault zone. (4) Owing to the tectonic and erosional history of the region, the topography is complex with high mountains and deep valleys. The height difference from the mountain tops to the valley floors is often more than 1000 m; in some cases, it is as much as 3000 m^18^. Snow and glaciers are often found at higher elevations, whilst more generally the terrain is complex and the climate is variable. These factors pose difficulties for direct investigations, sampling, geophysical exploration, and other field techniques. Owing to the steep surface slope (mostly ~ 45°) and the large amount of covered ground (e.g., by ice, snow, and vegetation), information acquired by satellite-based remote sensing is also often limited. Thus, under the influence of the above factors, research on karst groundwater in the eastern Qinghai–Xizang Plateau remains immature.

To perform research on a complex karst hydrological system, various technical methods should be applied to obtain as much information as possible^4,6,12^. First, using hydrochemical and isotopic methods to identify groundwater circulation can help improve understanding of the overall situation. It is difficult to directly describe the heterogeneity of karst aquifers based on hydrochemical data alone, whereas environmental isotopes (such as ^18^O and ^2^H) can provide additional information on the recharge characteristics of karst aquifers under different flow conditions^19–22^, specific groundwater flow paths^4,18^, and water transport times in karst aquifers^23–29^. Flow monitoring is also an essential method of investigation that can obtain long time series of flow information with which to examine dynamic characteristics^4^. Finally, satellite remote sensing is an effective approach for assessing the distribution, recharge, and discharge characteristics of glacial lakes^17^.

In this study, various technical methods were used to obtain data to elucidate the recharge source and circulation process of karst groundwater in the glacial lake area of the Jinsha River fault zone (China). To the best of our knowledge, this study represents the first attempt to use multisource data to investigate the karst groundwater circulation in the eastern Qinghai–Xizang Plateau. This paper aims to provide insight into the process of karst hydrology in the study region, and to provide reference data that could be useful in the study of karst aquifers with similar characteristics elsewhere, especially in areas with complex geological structures or distributed glacial lakes.

## Study Area

### Geographic and climatic setting

**Figure 1:**
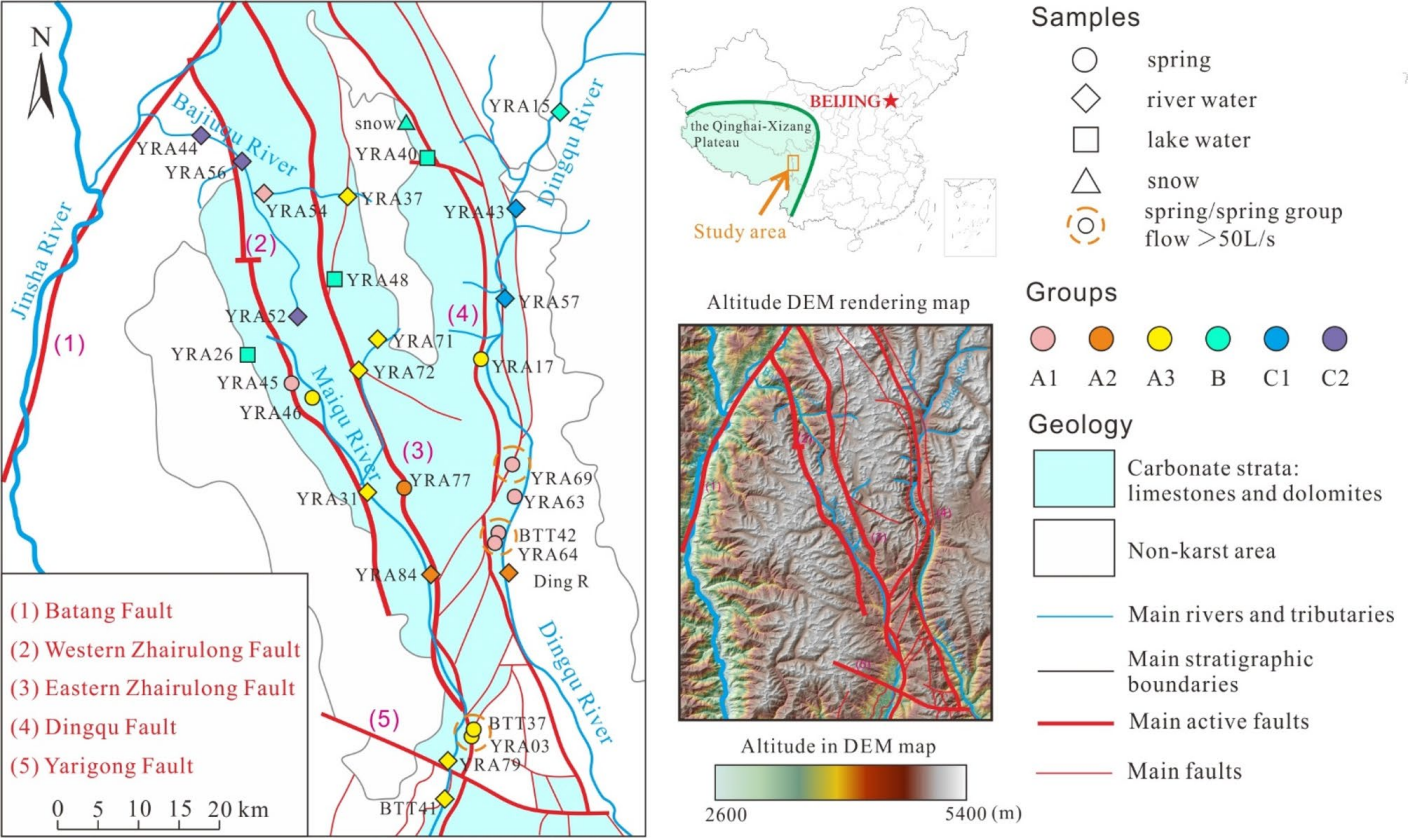
Hydrogeological map of the study area, including sampling locations. The faults, rivers, and area of carbonate rock distribution are from the China Geological Survey (https://www.cgs.gov.cn/), a digital elevation model rendering map background derived from the Spatial Data Cloud (http://www.gscloud.cn/) and generated using ArcGIS v 10.0, and a location map of the study area obtained from the Ministry of Natural Resources, People’s Republic of China (https://www.mnr.gov.cn/). The figure was generated using CorelDRAW X6.

The study area is located in the Hengduan Mountains in the east of the Qinghai–Xizang Plateau (Fig. 1). The highest peak in the area is located in the east of Batang County (5335 m above sea level; a.s.l.), and the lowest point is the Maiqu River valley (2950 m a.s.l.). Generally, the terrain is high in the north and lower in the south. The surface slope is widely ~ 45° but can reach up to 70° in some areas. Valleys typically present as V-shaped mountain canyon landforms with width of generally < 1000 m.

The regional climate can be classified as alpine-plateau type^30^, with annual average temperature of 14.8 °C, and average annual precipitation of 504 mm. The rainy season extends from June to September, during which approximately 90% of the total annual precipitation occurs.

The study area is on the eastern bank of the Batang section of the Jinsha River. The main rivers include the Bajiuqu River, which flows in a SE–NW direction, the Maiqu River, which flows in a N–S direction (along the Western Zhairulong Fault), and the Dingqu River, which flows along the Eastern Zhairulong Fault. All these rivers correspond to locations of karst groundwater discharge. Because no flow monitoring stations have been established in any of the rivers mentioned above, no flow monitoring data are available. According to direct measurements conducted in May 2021 the flow of the Bajiuqu River was 1.1 m^3^/s, that of the Maiqu River was 15 m^3^/s, and that of the upstream Dingqu River (measured at YRA43) was approximately 1.6 m^3^/s. The flow in the middle and downstream reaches of the Dingqu River could not be measured directly owing to excessively high flow.

Human settlements in the study area are concentrated near BTT37 and BTT42. Animal husbandry is one of the main human activities, and no industrial activities are in the region. Above 3500 m a.s.l., the environment is essentially undisturbed.

### Geology

Sedimentary rocks, dating from the Cambrian to the Carboniferous, are in the study area. The main carbonate rock formations are Cambrian-, Ordovician-, and Devonian-aged limestones, dolomites, and—to a lesser extent—marbles. The total thickness of the carbonate rock formations exceeds 1000 m. These formations mainly occur in the N–S striking block associated with the Western Zhairulong and Dingqu faults. The surrounding noncarbonate rocks are mainly sandstones, slates, and granites.

The study area is located within the Jinsha River fault zone at the intersection between the Eurasian and Indian Plates. The width of the Jinshajiang fault zone is more than 80 km, and its internal structure is highly complex. The age of the latest activity of the Batang Fault, Western Zhairulong Fault, and Eastern Zhairulong Fault is Holocene, while that of the Dingqu and Yarigong faults is late Pleistocene^31,32^. Of these, the Batang Fault has shown strong activity in the recent 150 years^33–35^. For example, an Ms7.2 earthquake occurred in 1870, an Ms6.7 earthquake occurred in 1989, and an Ms5.5 earthquake occurred in December 1996^36^.

These faults affect the occurrence of the strata, river locations, and certain landforms.

1. Under the control of the active faults, the area of distribution of the carbonate rocks is hourglass-shaped, with the thinner part in the middle of the hourglass aligned west–east through the Yarigong Fault.
2. The Dingqu River and the Maiqu River are distributed along the Dingqu Fault and the Western Zhairulong and Eastern Zhairulong faults, respectively.
3. Valleys are formed by the Dingqu River and the Maiqu River, and between the Dingqu and Eastern Zhairulong faults is a high-elevation area that is the main distribution area of glacial lakes.

### Karst development

According to^12^, karst systems within the study area occur mainly in three elevation bands: below 3500 m, 4000–4200 m, and > 5000 m. Nearly 70% of karst is developed around the faults.

Karst features in the region are mainly karst pores, karst gaps, and karst caves. Karst springs are mainly distributed below 3500 m a.s.l. Because the carbonate dissolution rate is slower than the downcutting rate of erosion in the river valleys, the karst springs mostly appear in the form of hanging springs. There are three springs with high flow (> 100 L/s): BTT37 + YRA03, BTT42 + YRA64, and YRA69.

### Glacial landforms

Since the late Cretaceous, strong crustal uplift and valley deepening have shaped the landform of high mountains and deep valleys within this area. According to^37^, the glacial landforms in the study area were formed from the late Pleistocene to the Holocene, coincident with a period of active tectonics. Glacial landforms such as angular peaks, glacial lakes, and U-shaped valleys are common between the Eastern Zhairulong Fault and the Dingqu Fault. Glaciers mostly moved along the stratigraphic fracture zone formed by tectonic activities, and many U-shaped valleys, small ice erosional platforms, and glacial lakes formed along the active faults.

Although most regional glaciers have melted, numerous glacial lakes remain. Under the influence of various actions such as glaciers and chemical dissolution, the current ice erosional and dissolution landforms of the plateau have formed^37–39^.

## Methods

### Remote sensing

QuickBird satellite data from April 2020, which have accuracy of 2–5 m, were selected (from https://discover.digitalglobe.com) to identify the locations and areas of the regional glacial lakes. First, preprocessing work such as atmospheric correction, image fusion, and stitching were performed using ENVI5.3 software. Then, interpretation of the glacial lakes was undertaken mainly through visual interpretation. To avoid influence from the shadows of surrounding mountains on the glacial lake classification, mountain shadows were first extracted, and then glacial lakes within the shaded areas were cross validated against multiperiod historical Google Earth images. Taking advantage of the similar spectral response characteristics of mountain shadows and glacial lakes, as well as their ultralow reflection in the near-infrared band, after many tests, the shadow extraction procedure adopted the values of the normalized difference water index (NDWI) of > 0.18 and the near-infrared spectrum of < 1000. Subsequently, glacial lakes were extracted based on a value of NDWI > 0.18. Taking into account the extraction accuracy and practical significance, the glacial lake extraction procedure excluded lakes with an area of fewer than 3 pixels.

To verify the correctness and accuracy of the satellite remote sensing lake extraction procedure, three accessible glacial lakes (i.e., YRA26, YRA40, and YRA48) were investigated in terms of parameters such as longitude and latitude, size, and lake depth.

### Flow measurement

The flow rates of the main karst springs (Fig. 1) in the study area were measured using the section-velocity method with an FP111 portable flow meter (Global Water, US). The flow meter range is 0.1–6.1 m/s, and its test accuracy is 0.03 m/s.

The flow measurements of all karst springs were undertaken during May 10–13, 2021 (dry season). Flow measurements were also conducted on karst springs BTT37, BTT42, and YRA69 on June 1, September 7, and November 17, 2021. Hydrometeorological conditions were fully considered during the flow measurements, and there was no precipitation within the study area during May 1–13. Thus, the current measurement data could be used in analysis of the same period.

A flow monitoring point at BTT37 was established on September 7, 2021. Karst spring flow is measured every 2 h. The automatic monitoring flow and the data measured in June, September, and November were checked to ensure the stability of the monitoring instrument.

### Sampling

Overall, 30 samples were collected: 10 from karst springs, 3 from glacial lakes, 15 from rivers, 1 was mountain-top snow (> 5200 m a.s.l.), and 1 was rainwater. All of the samples were collected during May 10–13, 2021, with the exception of the rainwater sample, which was collected on April 25, 2021. The locations of the sampling points are marked in Fig. 1.

The sampling and preservation processes adopted were based on^31^. Sampling bottles were rinsed with sample water at least three times before collection. The samples were filtered through 0.45-μm membrane filters and poured into 1.5-L and 250-mL high-density polyethylene bottles for analyses of major and trace elements. The 250-mL samples were acidified by adding double-distilled nitric acid until the pH was < 2; the 1.5-L samples were untreated. Samples were stored at 4 °C until analysis. Samples for stable isotope analysis (δ^18^O and δD) were collected in 50-mL glass bottles, which were sealed with airtight caps.

### Analytical methods

During sample collection, the water temperature, pH, and total dissolved solids (TDS) were measured using a HANNA HI 991301 pH/EC/Temperature multiparameter instrument (Hanna Instruments®, Woonsocket, RI, USA). The concentrations of K^+^, Na^+^, Mg^2+^, and Ca^2+^ were measured using a PerkinElmer Inductively Coupled Plasma Optical Emission Spectrometer (ICP-OES) Model Optima 8300 (precision: ± 1%) at the Groundwater Mineral Water and Environmental Monitoring Center of the Institute of Hydrogeology and Environmental Geology, Chinese Academy of Geological Sciences, Shijiazhuang, China. Major anions, except HCO_3_^−^, were analyzed using a Thermo Scientific Dionex ICS-4000 (precision: ± 1%); HCO_3_^−^ levels were determined via phenolphthalein titration. The reliability of the hydrochemical data was assessed by checking ion balances, which were found to be within ± 5%.

The δ^18^O and δD isotopes were determined using a Picarro L2130-i Analyzer at the Institute of Hydrogeology and Environmental Geology, Chinese Academy of Geological Sciences. The data were expressed in delta notation (δ) as parts per thousand (‰) relative to the Vienna Standard Mean Ocean Water (V-SMOW), with precision of ± 0.1‰ for δ^18^O and ± 1‰ for δD.

Tritium samples were analyzed using an ultralow background liquid scintillation spectrometer (1220 Quantulus) after low-temperature electrolysis enrichment. The detection limit and test precision were 1 and ± 0.5 TU, respectively. Tritium was used to determine the mean age of the groundwater. According to the classification method of the International Atomic Energy Agency, when the tritium content is < 3 TU, the groundwater might be derived from recharge prior to 1954; when the tritium content is 3–20 TU, the groundwater is likely to have been recharged between 1954 and 1961. Some researchers also consider that if the tritium content is n × 10^0^ TU (where n is a natural number), it indicates mixed water (i.e., mixed stagnant water and n × 10^0^ to n × 10^2^ TU water), while n × 10^1^ TU indicates a mixture of recent precipitation and n × 10^2^ TU water^40^.

## Results

### Distribution of glacial lakes

**Figure 2:**
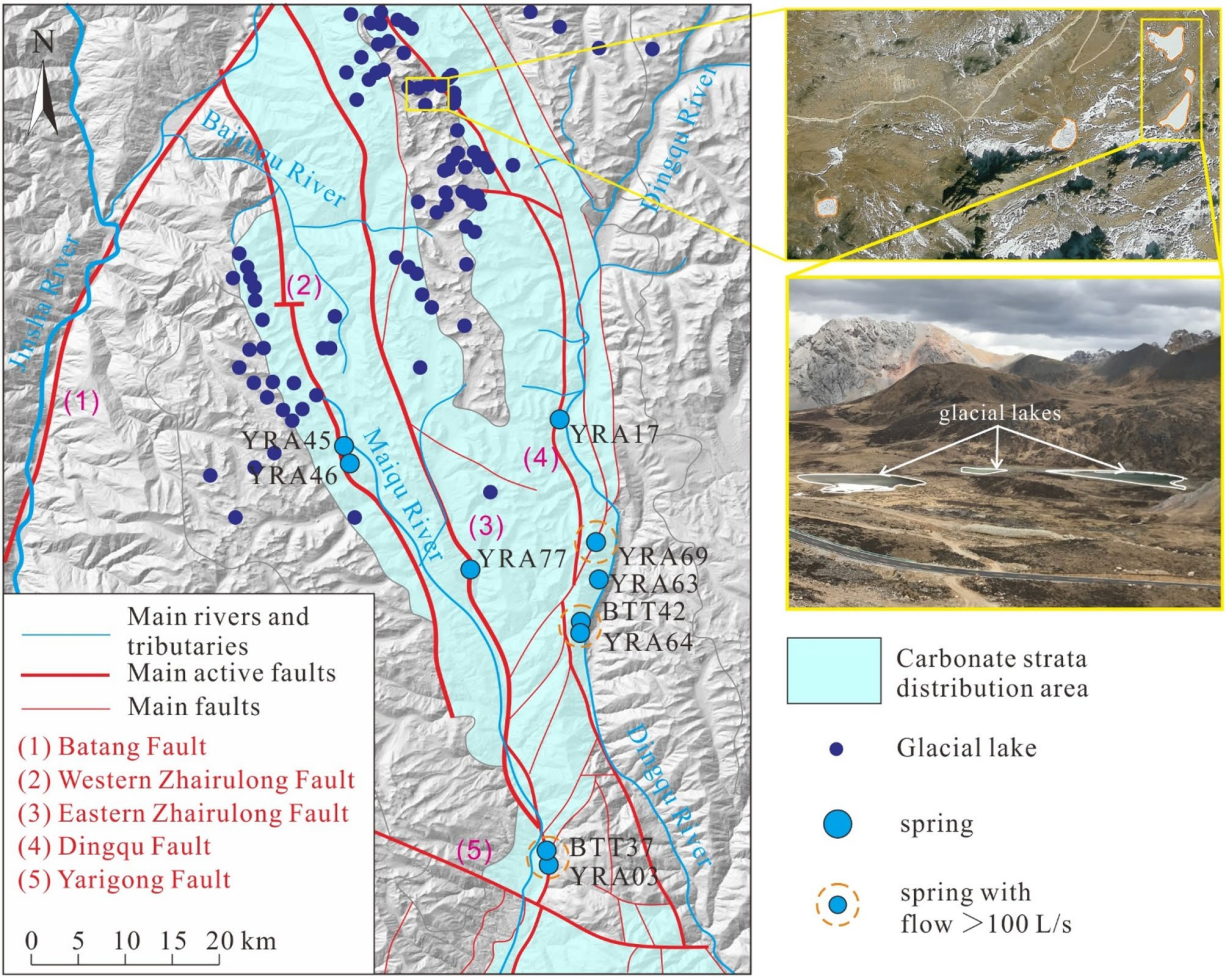
Distribution of glacial lakes in the study area. The digital elevation model rendering map background was derived from the Spatial Data Cloud (http://www.gscloud.cn/) and generated using ArcGIS v 10.0. The figure was generated using CorelDRAW X6.

A total of 176 lakes are distributed within the study area, and most of them (143/176) are located in the 4400–5000 m elevation band between the Western Zhairulong Fault and the Dingqu Fault (Fig. 2). The total glacial lake area across the region is nearly 5.12 km^2^, with individual lake surfaces generally in the range of 0.1–5.0 × 10^4^ m^2^ (141/176). The maximum (minimum) lake area is 200.2 × 10^4^ m^2^ (0.03 × 10^4^ m^2^), and the maximum water depth of all accessible glacial lakes is approximately 0.4–1.2 m.

Lake surface shapes are mostly irregular and approximate ellipses, the long axes of which mostly align with the strike direction of nearby faults. At most lakes, only the inputs of meltwater and rainfall can be observed, while no “outlet” can be observed. Typically, the rock mass around the lakes is weathered, and rock fissures and solution fissures are strongly developed.

### Spring flows

Table 1 shows that the elevation of the karst springs in the study area is between 3200 and 4550 m a.s.l. Three karst springs have large flow (> 100 L/s): BTT37 + YRA03 (143 L/s in total), BTT42 + YRA 64 (133 L/s in total), and YRA 69 (216 L/s). The combined flows of these three karst springs account for approximately 59% of the total flow of regional karst springs (823 L/s), meaning that there are runoff conduits of karst groundwater within the study area. These three groups of karst springs emerge at elevations between 3200 and 3500 m a.s.l., indicating that this is the elevation of regional karst water discharge. Horizontally, they are exposed near active faults, indicating that the active faults control the flow and discharge of karst water in the study area.

**Table 1:** Flow of the main karst springs within the study area.

	flow(L·s^−1^)	a.s.l.(m)
BTT37	78	3276
BTT42	126.9	3454
YRA03	64.94	3265
YRA17	17.55	3670
YRA45	10.41	4211
YRA46	2.8	4289
YRA63	23.8	3498
YRA64	6.2	3432
YRA69	216.1	3487
YRA77	8.2	3874
Total	829	

**Figure 3:**
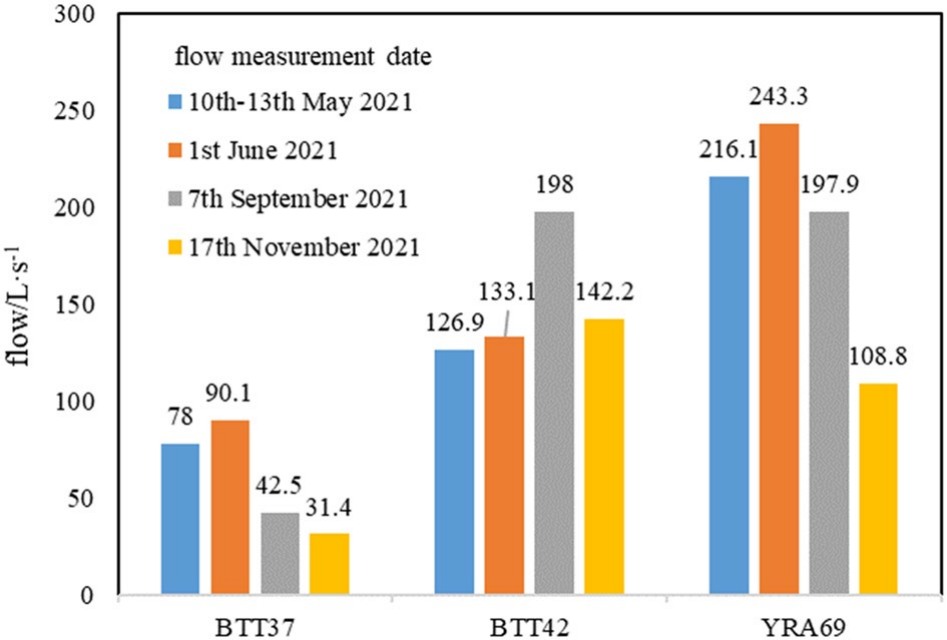
Flow of main karst springs within the study area.

**Figure 4:**
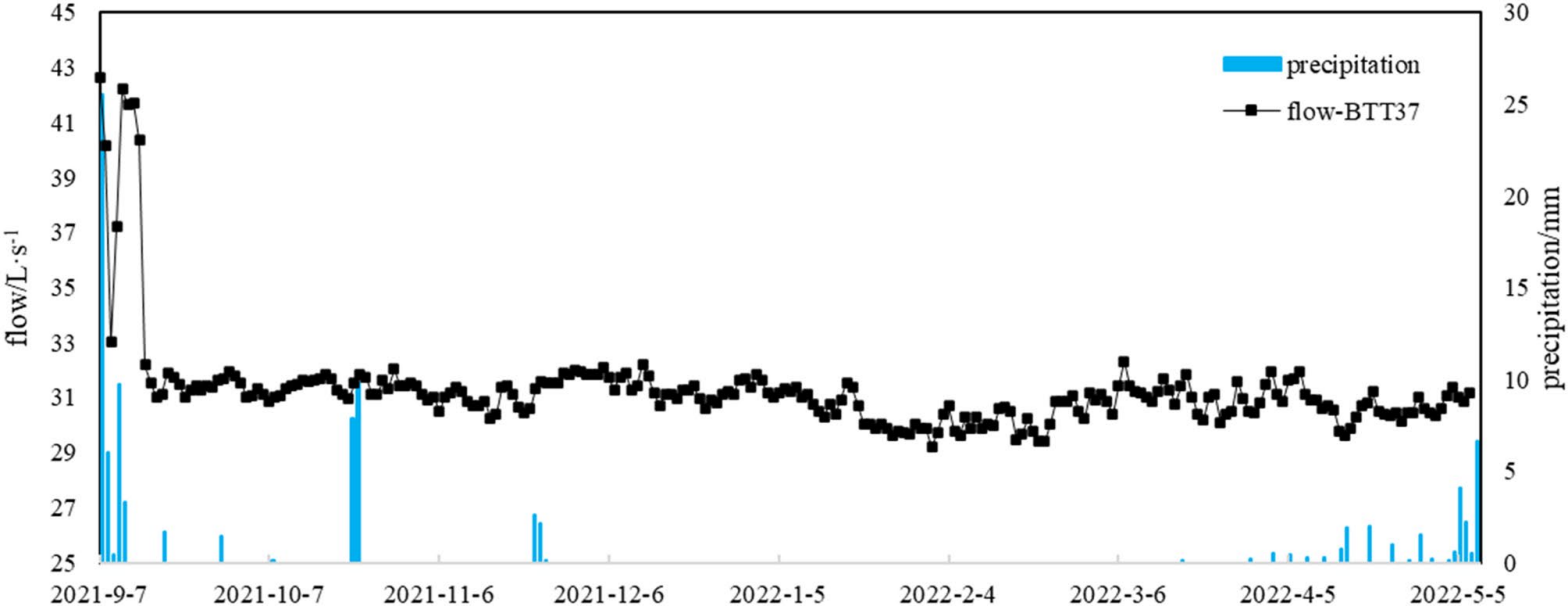
Precipitation (measured at the Batang meteorological station, data from http://data.cma.cn/)) and flow of BTT37 from September 7, 2021 to May 5, 2022.

Figure 3 shows the flow of karst springs BTT37, BTT42, and YRA69 measured in May, June, September, and November 2021. The discharge variation coefficients (ratio of the standard deviation to the average) of the three karst springs are 0.40 (BTT37), 0.19 (BTT42) and 0.26 (YRA69). Springs BTT37 and YRA69 have the same flow change law; that is, the highest in June, second highest in May, and lowest in November. Spring BTT42 has the highest flow in September, and its flow in the other months is broadly consistent at 125–142 L/s. Figure 4 shows that the flow of BTT37 has changed little after September 14. In the dry season of 2021, the flow of BTT37 did not diminish notably, and since April 2022, despite the increase in precipitation, the flow has not increased substantially.

### Hydrochemical and isotopic water composition

The pH values of waters sampled from the glacial lakes, main karst springs, and rivers show some variability (Table 2). The pH value of glacial lake water is 7.35–7.44 (i.e., nearly neutral), which is similar to that of snow (7.38). The pH values of the main karst springs and river water are 7.50–8.16 and 7.68–8.32, respectively; that is, neutral or weakly alkaline.

**Table 2:** Hydrochemical types and major ion concentrations of samples from within the study area.

sample ID	sample type^1)^	pH	EC (μS/cm)	TDS (mg/L)	Na^+^ (mg/L)	K^+^ (mg/L)	Mg^2+^ (mg/L)	Ca^2+^ (mg/L)	Cl^-^ (mg/L)	SO_4_^2-^ (mg/L)	HCO_3_^-^ (mg/L)	Water type
BTT37	GW	7.95	219	106	0.23	0.12	6.37	31.55	< 0.10	0.65	126.4	Ca·Mg–HCO_3_
BTT42	GW	7.8	372	180	3.69	0.6	10.19	47.85	0.19	8.08	192.9	Ca·Mg–HCO_3_
YRA03	GW	7.94	253	117	0.45	0.21	7.86	32.2	< 0.10	1.68	133	Ca·Mg–HCO_3_
YRA17	GW	7.89	191	90	0.25	0.16	7.44	22.06	< 0.10	1.35	102.8	Ca·Mg–HCO_3_
YRA45	GW	8.16	371	184	0.9	0.51	11.12	51.54	< 0.10	12.2	193.3	Ca·Mg–HCO_3_
YRA46	GW	7.5	239	115	1.58	0.61	5.82	31	< 0.10	13.54	108.7	Ca·Mg-HCO_3_
YRA63	GW	7.87	350	173	3.56	0.63	10.16	45.92	0.17	9.04	184.2	Ca·Mg-HCO_3_
YRA64	GW	8	354	175	3.72	0.63	10.19	46.51	0.15	8.09	187.3	Ca·Mg-HCO_3_
YRA69	GW	7.71	429	207	5.75	0.91	10.86	54.83	0.3	9.15	223.5	Ca·Mg-HCO_3_
YRA77	GW	7.78	325	155	0.93	0.5	8.66	43.22	1.4	6.42	168.3	Ca·Mg-HCO_3_
YRA26	LK	7.38	91	45	1.74	0.6	1.19	9.26	0.2	6.38	30.84	Ca-HCO_3_·SO_4_
YRA40	LK	7.35	140	69	0.97	0.51	2.83	18.39	0.1	6.08	66.45	Ca-HCO_3_
YRA48	LK	7.44	19	9	0.25	0.08	0.1	1.37	0.15	< 0.20	12.08	Ca-HCO_3_
BTT41	RW	8.09	263	130	0.78	0.39	10.17	32.88	< 0.10	10.63	133.6	Ca·Mg-HCO_3_
YRA15	RW	7.68	114	52	3.35	0.58	0.84	10.65	< 0.10	7.58	36.28	Ca-HCO_3_·SO_4_
YRA31	RW	7.79	189	92	1.09	1.17	2.86	25.68	< 0.10	5.51	90.01	Ca-HCO_3_
YRA37	RW	7.76	230	111	1.12	0.58	6.18	29.77	< 0.10	13.39	102.7	Ca·Mg-HCO_3_
YRA43	RW	7.87	504	251	1.91	0.91	8.81	68.62	< 0.10	91.31	138.9	Ca-HCO_3_·SO_4_
YRA44	RW	8.04	425	203	8.64	1.44	15.75	45.11	0.97	19.07	204.8	Ca·Mg-HCO_3_
YRA52	RW	8.32	417	206	4.52	0.84	25.87	36.35	0.24	15.9	199.3	Mg·Ca-HCO_3_
YRA54	RW	8.13	325	159	5.8	1.07	11.67	36.67	0.44	11.2	166.1	Ca·Mg-HCO_3_
YRA56	RW	8.04	568	277	7.45	2.02	33.64	43.53	0.49	34	268.8	Mg·Ca-HCO_3_
YRA57	RW	8.16	463	228	2.1	1.5	11.83	55.56	< 0.10	78.44	132.9	Ca·Mg-HCO_3_-SO_4_
YRA84	RW	7.8	285	141	4.22	2.6	8.39	31.62	0.51	2.97	148	Ca·Mg-HCO_3_
YRA72	RW	7.92	249	124	0.38	0.62	4.03	38.23	< 0.10	0.92	138.9	Ca-HCO_3_
YRA71	RW	8.06	238	113	0.57	0.24	3.22	36.08	< 0.10	1.8	126.9	Ca-HCO_3_
YRA79	RW	8.09	297	143	0.64	0.39	5.22	45.54	0.11	1.02	169.1	Ca-HCO_3_
Ding R	RW	7.92	193	95	1.61	0.78	3.13	25.88	1.05	20.87	66.73	Ca-HCO_3_·SO_4_
Snow	snow	7.38	23	10	0.09	0.08	0.05	2.11	0.35	1.5	11.42	Ca-HCO_3_
Rain-Apr	rain	7.7	20	9	0.33	0.17	0.18	1.31	0.21	0.3	12.08	Ca-HCO_3_

1) Sample type, GW—groundwater, LK—lake, RW-river water.

The TDS values of the different types of samples align with theory, with snow and rain TDS concentrations (9–15 mg/L) lower than glacial lake TDS concentrations (9–69 mg/L), which in turn are lower than river water TDS concentrations (52–277 mg/L). River water concentrations of TDS are similar to those of groundwater (90–207 mg/L).

**Figure 5:**
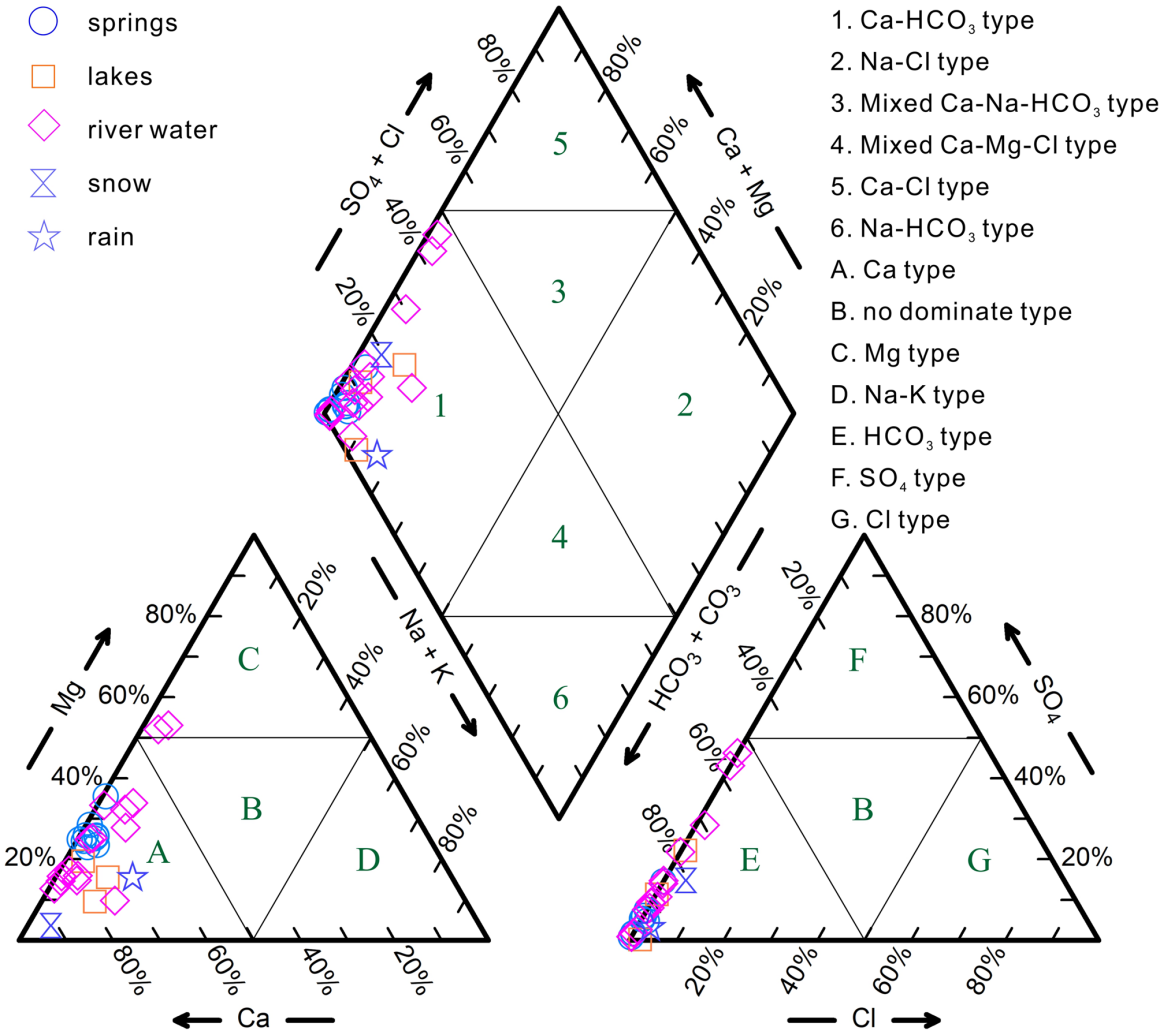
Piper diagram of water samples from within the study area.

The main cations of all samples are Ca^2+^ and/or Mg^2+^ (Fig. 5). The main anion is HCO_3_^−^, and the SO_4_^2−^ concentration of some river water (YRA15, YRA43, and YRA57) is slightly higher than that of other samples, which might be related to the impacts of agricultural production and animal husbandry activities.

The stable isotope values of spring water samples ranged from − 19.5 to − 18.2‰ for ^18^O, with a mean value of − 18.9‰, and from − 146 to − 138‰ for δD, with a mean value of − 142.5‰ (Table 3). Using the least squares method^24^, the relationship between δD and δ^18^O was calculated as follows:

1 $$ \delta D = 6.299 \times \delta^{18} O - 23.503\;\left( {{\text{R}}^{{2}} = 0.{91}} \right) $$

**Table 3:** Measurements of δD and δ^18^O in water samples from the study area.

	δD (‰)			δ^18^O (‰)		
	Max	Min	Average	Max	Min	Average
Springs	− 138.0	− 146.0	− 142.5	− 18.2	− 19.5	− 18.9
Lakes	− 127.0	− 136.0	− 131.3	− 16.6	− 17.9	− 17.3
River Water	− 133.0	− 144.0	− 138.7	− 17.4	− 18.8	− 18.3
Snow	− 177.0			− 24.1		
Rain	− 79.0			− 10.0		

The isotopic values of the glacial lake water samples varied between − 17.9 and − 16.6‰ for δ^18^O (mean value: − 17.3‰), and between − 136 and − 127‰ for δD (mean value: − 131.3‰). The isotopic values of the river water samples were between − 18.8 and − 17.4‰ for δ^18^O (mean value: − 18.3‰), and between − 144 and − 133‰ for δD (mean value: − 133‰). As such, the river water appears more isotopically depleted than the lake water. Values of δ^18^O and δD in the samples of snow and rain were higher than those in the samples of spring, lake, and river water.

**Figure 6:**
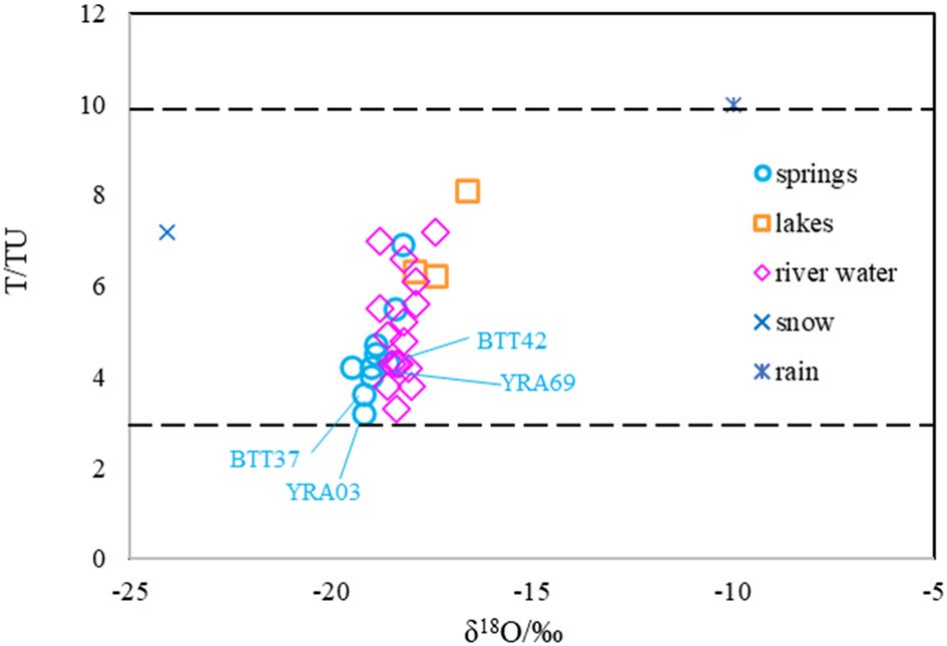
Plot of δ^18^O vs. T values of water samples. Karst springs with flow of > 50 L/s are marked.

All the tritium values of the samples of glacial lake water, river water, and groundwater were between 3 and 10 TU (Fig. 6), indicating that the groundwater is mainly derived from the mixing of lake water and modern precipitation. The river water is mainly supplied by groundwater discharge. The tritium values of the three groups of karst springs with high flow are relatively low (< 4.7 TU), indicating long residence time of groundwater in the aquifer.

## Discussion

### Water origins

The hydrochemical types of the water samples are similar. However, the law of TDS values (Table 2) of the different types of water samples suggest that snow and rain might be the initial water sources, and that glacial lakes and groundwater are recharged in turn before discharge occurs into rivers. Water–rock interaction occurs during flow through the subsurface karst system.

**Figure 7:**
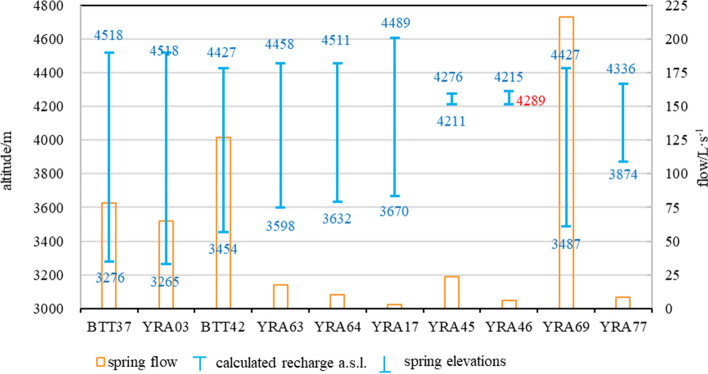
Calculated elevation of recharge areas, spring elevations, and flow of karst springs within the study area.

The isotopic composition of groundwater in mountainous areas is often affected by elevation effects, which depend on the elevation of the groundwater recharge area. According to Yao et al.^41^, the oxygen isotopic composition of atmospheric precipitation on the Qinghai**–**Xizang Plateau exhibits the following relationship:

2 $$ \delta^{18} O = - 0.0033h - 4.29, $$

where *h* is elevation (m). According to Eq. (2), the average recharge elevation of the main karst springs is 4424 m (Table 4). The recharge area of the karst springs with flow of > 50 L/s is located above 4400 m a.s.l. (Fig. 7), which is the same as the distribution elevation of the glacial lakes as mentioned in Section “Distribution of glacial lakes”. Thus, the elevation of the main karst spring recharge area coincides with that of the glacial lake distribution. For karst springs with flow of > 50 L/s, the average elevation difference between the recharge area and the discharge area is 1102 m, which is greater than that of other karst springs. With consideration of the data that show that the combined flows of these springs account for 59% of the total karst spring flow within the study area, it may be concluded that these are the main conduits of karst groundwater flow. Groundwater in the main runoff conduits has a long flow path and it forms large karst springs (i.e., karst springs with relatively high flow).

**Table 4:** Spring elevation and measured data of discharge areas for groundwater samples.

Sample ID	calculated recharge a. s. l/m	spring elevation/m	vertical distances/m
BTT37	4518	3276	1242
YRA03	4518	3265	1253
BTT42	4427	3454	973
YRA63	4458	3598	960
YRA64	4458	3632	1026
YRA17	4609	3670	939
YRA45	4276	4211	65
YRA46	4215	4289	−74*
YRA69	4427	3487	940
YRA77	4336	3874	462

*: the negative value is due to the possible fitting deviation in formula (1), and indicates that the altitude difference between the recharge area and discharge area of karst spring numbered YRA46 is not large.

Other karst springs can be divided into two groups: karst springs with vertical circulation distance of 900–1000 m and flow rate of approximately 20 L/s (YRA17, YRA63, and YRA64) and epikarst springs with short vertical flow distance (YRA45, YRA46, and YRA77).

According to Song et al.^42^, precipitation in the study area originates in the Indian Ocean, Pacific Ocean, and Central Asia. Meanwhile, the water vapor source of the local internal circulation is also important, and the short cycle leads to relatively insignificant fractionation effects^43^.

**Figure 8:**
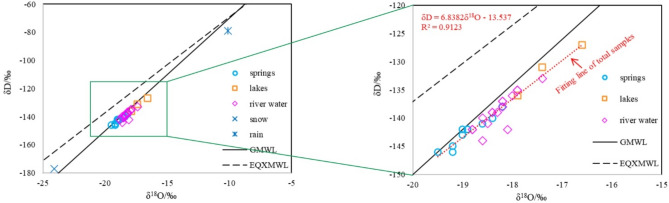
Plot of δ^18^O versus δD. The GMWL and EQXMWL are also shown.

During water vapor transport, isotopic fractionation occurs, resulting in depletion of δD and δ^18^O in the study area. Owing to the topography of high mountains and deep valleys, the study area is dominated by the local water cycle. The stable isotope signatures and the fitting line of all karst springs fall near the global meteoric water line (GMWL) and far from the eastern Qinghai–Xizang Plateau meteoric water line (EQXMWL) (Fig. 8), which provides evidence for the atmospheric origin of these waters.

**Figure 9:**
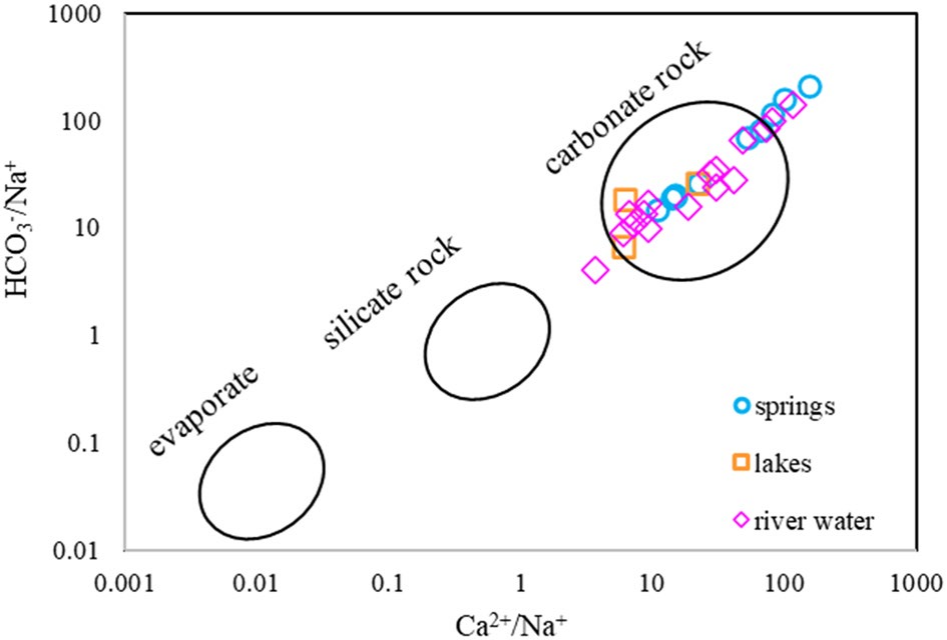
Plots of the relationship between HCO_3_^−^/Na^+^ and Ca^2+^/Na^+^ in groundwater. The circles and ellipses are schematic representations of typical water–rock interactions.

Glacial lake water, river water, and karst springs within the study area are supplied by local precipitation and ice meltwater ($\delta D = 6.84\delta^{18} O - 13.537$, R^2^ = 0.9123; Fig. 9). The δD and δ^18^O values in the glacial lake water are higher than those in the karst spring water, indicating that δD and δ^18^O are depleted in the runoff process. Values of δD and δ^18^O in river water are located between those of lake water and karst water because river water is the ultimate output of glacial lake water and karst water within the study area.

### Glacial lakes and groundwater flow dynamics

Average annual precipitation in the study area is 504 mm. According to the calculation of a carbonate area of 1249 km^2^, the annual precipitation volume is 629.77 × 10^6^ m^3^. Additionally, according to an assumed rainfall infiltration coefficient of 0.01–0.30 in karst areas^44^, the total amount of karst groundwater is 19.16–159.63 × 10^6^ m^3^. The measured karst spring flow (Table 2) is 26.14 × 10^6^ m^3^/a. Meanwhile, the TDS value of all karst springs is 90–207 mg/L, and the tritium content is 3.2–6.9TU, indicating that the karst groundwater circulation rate is rapid and that those flow paths are short. The above two points suggest that there is no water input from external sources.

As mentioned above, more than 90% of the annual precipitation in the study area occurs between June and September. In groundwater systems that mainly depend on precipitation supply and rapid runoff, spring flow fluctuations should quickly reflect precipitation fluctuations. Although there will be a certain lag, the overall flow should exhibit the same pattern of higher flow in the rainy season and lower flow in the dry season. However, Fig. 4 shows that the flow of the BTT37 remained stable and did not fluctuate substantially with atmospheric precipitation (even after October onward, the study area is covered with snow, with less precipitation and less ice meltwater). The flow variation law and flow variation coefficient of BTT42 and YRA69 are similar to those of BTT37 (Fig. 3), indicating that the flow variation characteristics of the main karst springs in the study area should be similar.

Two explanations may be given for this phenomenon. First, sources of groundwater recharge might be stable and constant. Second, the spring water recharge area is relatively large and the flow path distances to each spring are different. Hence, the flow peaks brought by precipitation events arrive at the springs at different times, and the superposition effect of spring flow peaks and troughs leads to an overall small fluctuation of spring flow. Compared with the Jakham River basin^45^, Niangziguan karst area^2^, Heilongdong spring basin^46^, and other areas, the catchment area of the karst water in the study area is not large; therefore, the first reason has greater plausibility.

Given the karst water recharge elevation calculation results, we consider it likely that glacial lakes play a major role in resource regulation and storage, receiving a large amount of recharge in the rainy season, and recharging the karst springs in the dry season (even in winter, the lakes still maintain a certain extent of karst groundwater recharge).

The origin and distribution of glacial lakes are related to active faults. The fracture zones formed by tectonic activity can form conduits for lake water infiltration and recharge. A large amount of lake water continuously and steadily infiltrates into karst conduits through active faults. Around the active faults, the degree of karst development is generally high^12^. The flow of groundwater is along the main runoff zone formed by the Eastern Zhairulong Fault and the Dingqu Fault, and is controlled by the drainage base level, insoluble rock strata, or water-blocking faults (e.g., the Yarigong Fault). In this process, owing to the large height difference between the recharge area and the discharge area, and the high development of fissures and karst conduits around the active fault, runoff is rapid; therefore, there is little “old” groundwater (such that the tritium values of all karst springs are > 4 TU). Indeed, there are also many epikarst springs with small flow and large flow fluctuation whose recharge and runoff are mainly controlled by terrain.

### Water–rock interactions

**Figure 10:**
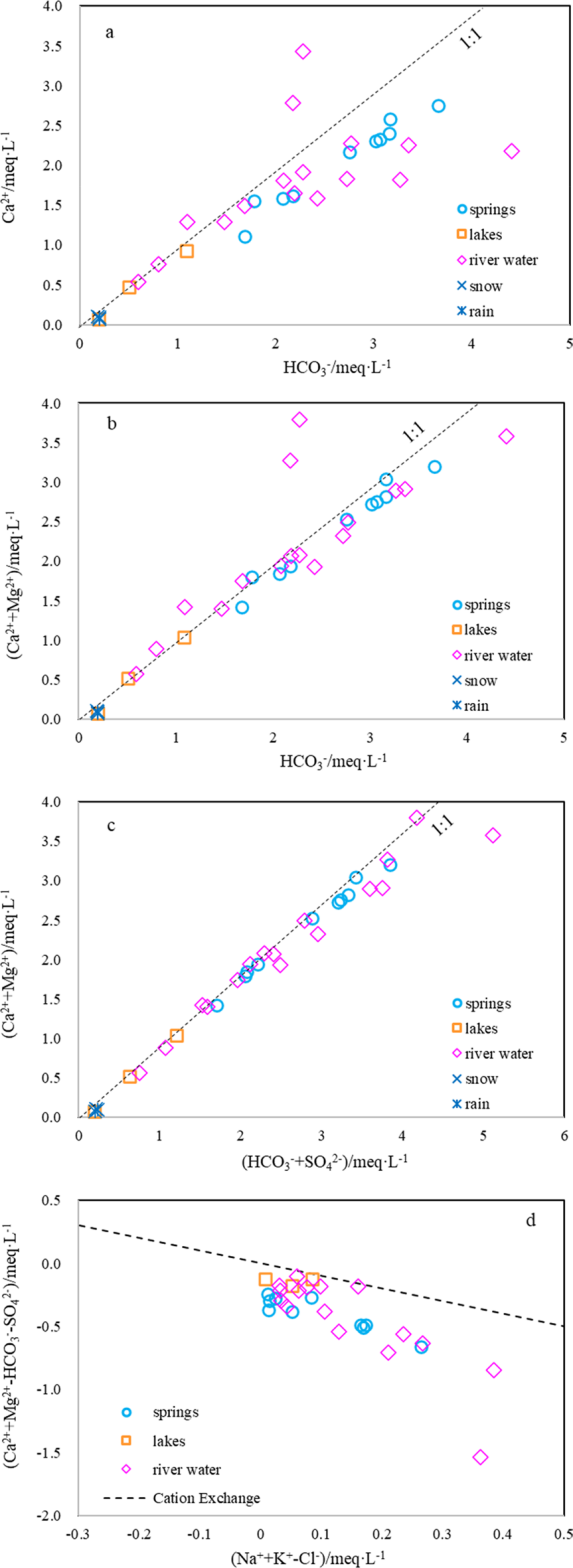
Ionic relationships of the main water groups in the study area: (**a**) HCO_3_^-^ vs. Ca^2+^, (**b**) HCO_3_^-^ vs. Ca^2+^ + Mg^2+^, (**c**) HCO_3_^−^ + SO_4_^2−^ vs. Ca^2+^ + Mg^2+^, and (**d**) Na^+^ + K^+^–Cl^−^ vs. Ca^2+^ + Mg^2+^–HCO_3_^–^SO_4_^2-^.

The chemical composition of water is mainly dependent on the dissolution of carbonate rock (Fig. 9). The main ionic components that arise from the dissolution of calcite and dolomite are illustrated in Fig. 10a and b. The source of SO_4_^2−^ is mainly the dissolution of gypsum, and might be associated with agricultural and animal husbandry (the river water point in the upper-right corner of Fig. 10c is offset from the 1:1 line).

Figure 10d, which shows the relationship between Na^+^ + K^+^–Cl^−^ and Ca^2+^ + Mg^2+^–SO_4_^2−^HCO_3_^−^, shows that almost all points are far from the cation exchange line, indicating little cation exchange during subsurface flow.

### Groundwater runoff path

**Figure 11:**
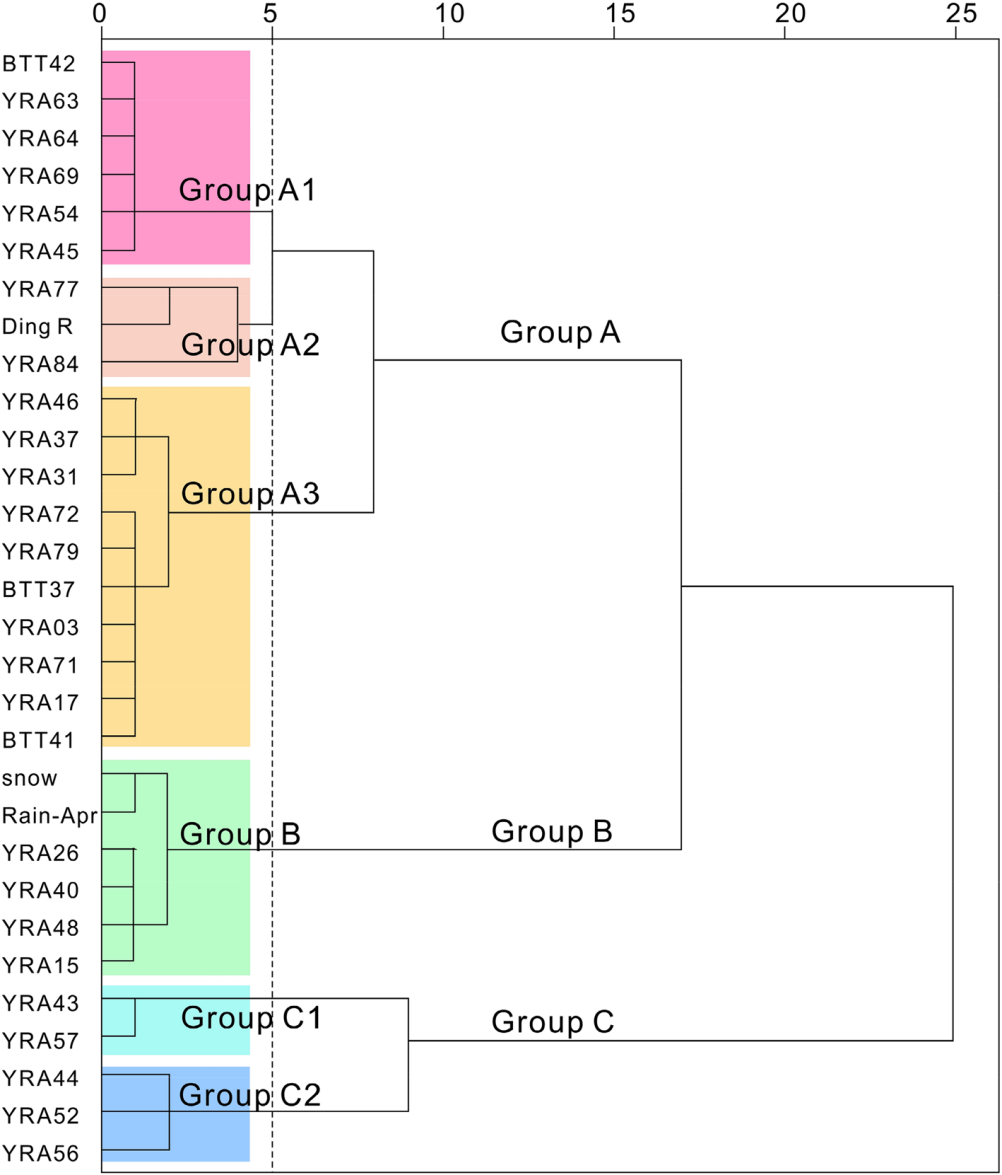
Dendrogram of the hierarchical cluster analaysis showing the identified groups and subgroups.

The hydrochemical characteristics of the water samples obtained in the study area are similar (Fig. 5). For further analysis of groundwater runoff path, hierarchical cluster analysis (HCA) was applied using Ward’s linkage to classify the samples and Euclidean square distances were taken for similarity measurements. The hydrochemical data (Ca^2+^, Mg^2+^, Na^+^, K^+^, HCO_3_^−^, SO_4_^2−^, Cl^−^, EC, pH, and TDS) of each water sample were standardized as input variables in the analysis. The HCA results are presented as a dendrogram in Fig. 11.

According to the characteristics of the dendrogram, the samples from the study area were classified into three major groups (A, B, and C). Additionally, groups A and C were further composed of three and two subgroups, respectively (A1, A2, and A3 and C1 and C2). The spatial distributions of the groups defined by HCA are shown in Fig. 1, and the characteristics of the water types are summarized in Table 5.

**Table 5:** Characteristics of the water types of the groups derived through hierarchical cluster analaysis.

groups	Water type	pH	EC	TDS
A	Ca–HCO_3_	7.9	283	137.37
A1	Ca·Mg–HCO_3_	7.9	367	179.67
A2	Ca·Mg–HCO_3_	7.8	268	130.33
A3	Ca–HCO_3_	7.9	237	114.10
B	Ca–HCO_3_	7.5	68	32.33
C	Ca/Mg–HCO_3_	8.1	475	233.00
C1	Ca–HCO_3_/HCO_3_·SO_4_	8.0	483	239.50
C2	Mg·Ca–HCO_3_	8.1	470	228.67

Group A1 contains six samples (five karst spring samples and one river water sample) with the hydrochemical type Ca·Mg–HCO_3_, which is primarily affected by water–rock interaction. The average TDS concentration in this group is 179.67 mg/L.

Group A2 contains two river water samples and one karst spring sample from a high-elevation spring (YRA77, 3874 m). The pH value and the hydrochemical type are similar to those of Group A1, but the TDS values are lower.

Group A3 contains 33% of all samples, including most karst spring samples and some river water samples. Moreover, most samples of this group were collected in the Maiqu River basin. The hydrochemical type is mainly Ca-HCO_3_ and the mean TDS value is 114.10 mg/L; that is, lower than that of Groups A1 and A2.

In Group B, the water samples have characteristics of low pH and TDS values. The samples comprise three glacial lake samples, one river water sample collected in the source area of the Dingqu River, a snow sample, and a rainfall sample. All samples represent a water source or are from locations in a source area, and have not undergone water–rock interaction.

Groups C1 and C2 have regional characteristics. Group C1 contains two river water samples collected in the pastoral area of the Dingqu River basin, and C2 represents water collected from main stream of the Bajiuqu River.

According to the HCA results, most karst springs in Group A1 are located on the west bank of the Dingqu River, and most karst springs in Groups A2 and A3 are located in the Maiqu River basin (Fig. 1), showing that the karst groundwater of Group A1 has a different runoff path to that of Groups A2 and A3. Moreover, the low salinity of Group A indicates short residence time of groundwater in the aquifer.

**Figure 12:**
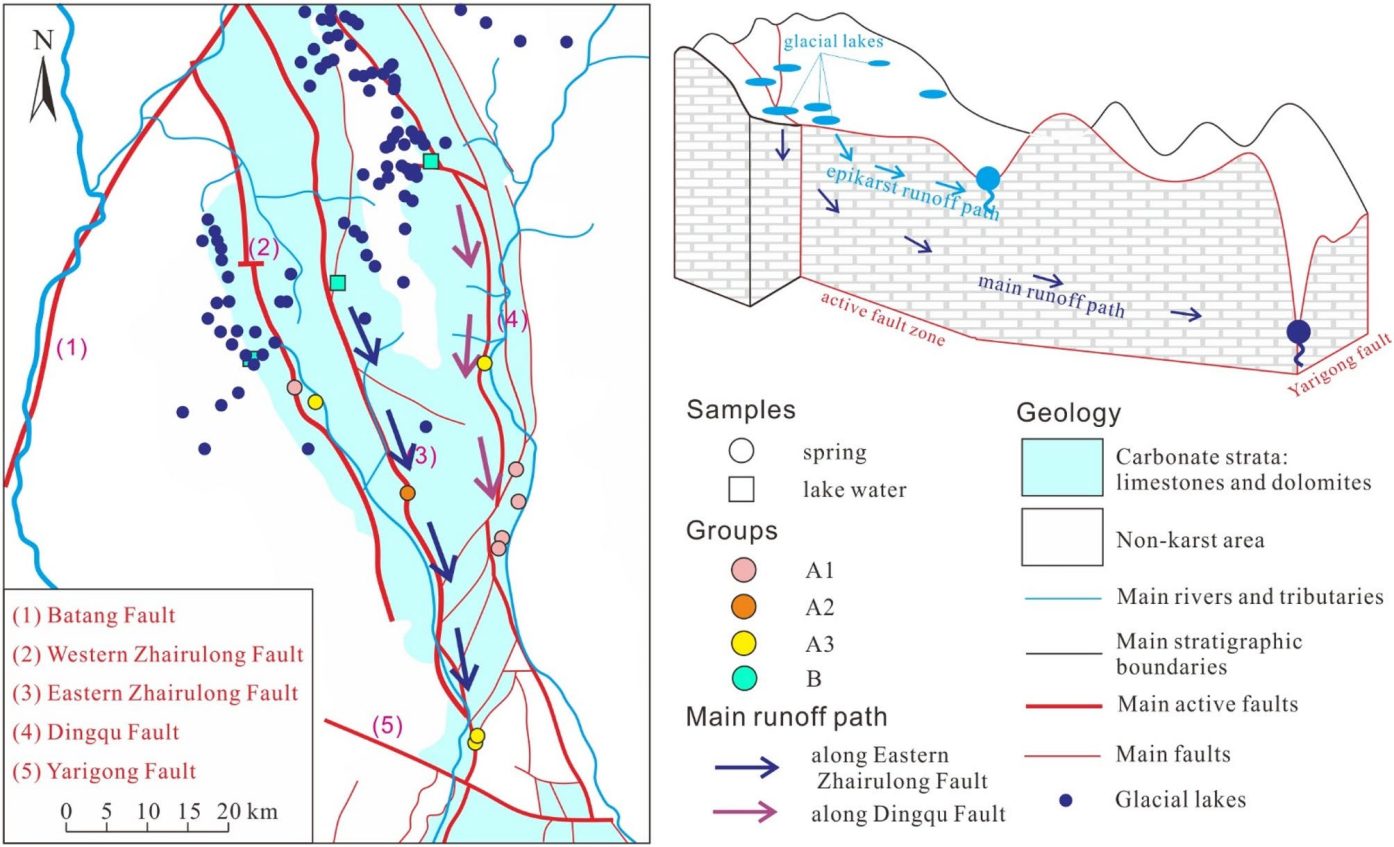
Recharge–runoff conceptual diagram and main runoff path of karst groundwater. The faults, rivers, are area of carbonate rock distribution are from the China Geological Survey (https://www.cgs.gov.cn/). The figure was generated using CorelDRAW X6.

The area of distribution of carbonate rocks in the study area is hourglass-shaped and the karst springs are mainly located in the thinner part in the middle of the hourglass. The mountains are steep, the area receiving precipitation is small, and there is no distribution of glacial lakes. Therefore, the supply of karst water mainly comes from the area of glacier lakes in the northern part of the study area. In the eastern Qinghai–Xizang Plateau, most of the karst is developed along faults, and two runoff conduits of karst groundwater are formed along the active Dingqu Fault and Eastern Zhairulong Fault. Groundwater flows from north to south along the groundwater runoff channel, is affected by the Yarigong Fault in the middle of the hourglass, and is discharged in the form of karst springs (represented by karst springs with flow of > 100 L/s, e.g., BTT37 + YRA03, BTT42 + YRA64, and YRA69) (Fig. 12).

Owing to the large height difference between the recharge area and the discharge area (Fig. 7), the runoff speed is fast (Fig. 6). In the process of runoff, the main ionic components arise from the dissolution of carbonate rock (Fig. 9) and cation exchange is weak (Fig. 10).

There are also some surface karst springs in the study area, and their circulation process is mainly controlled by topographic and geomorphic conditions.

## Conclusions

In this study, the origin and runoff of karst groundwater in the area of glacial lakes in the Jinsha River fault zone were investigated through analysis of multisource data, and the following conclusions were derived.

1. Glacial lake water is an important source of karst groundwater in the Jinsha River fault zone. Stable isotope data indicate that the recharge elevation is above 4400 m a.s.l., which coincides with the elevation of distribution of the glacial lakes. Lakes, which serve to regulate and store regional water resources, can continuously recharge karst water through fault fracture zones and karst conduits, thereby maintaining the relative stability of karst spring flows.
2. Active faults affect the runoff and discharge of karst groundwater in the eastern Qinghai–Xizang Plateau. Through HCA, karst groundwater runoff conduits were identified along two active faults: the Dingqu Fault and the Eastern Zhairulong Fault, which account for 59% of the total karst groundwater flow.
3. The vertical distance between the recharge area and discharge area of the three groups of karst springs with large flow is > 1000 m. The groundwater runoff is fast and the residence time in the aquifer is short. Dissolution of calcite and dolomite mainly occurs in the runoff process, and cation exchange is weak. Therefore, karst springs are mainly of HCO_3_–Ca·Mg type.

The results also show that the glacial lakes in the eastern Qinghai–Xizang Plateau have important maintenance functions regarding karst groundwater resources and the ecological environment, which need continuous monitoring and protection.

## Data Availability

The data used to support the findings of this study are available from the corresponding author upon request.
